# Investigating the Functional Roles of Aldehyde Dehydrogenase 3A1 in Human Corneal Epithelial Cells

**DOI:** 10.3390/ijms24065845

**Published:** 2023-03-19

**Authors:** Georgia-Persephoni Voulgaridou, Vasileios Theologidis, Maria Venetikidou, Ilias Tsochantaridis, Avgi Tsolou, Maria Koffa, Mihalis I. Panayiotidis, Aglaia Pappa

**Affiliations:** 1 Department of Molecular Biology and Genetics, School of Health Sciences, Democritus University of Thrace, 68100 Alexandroupolis, Greece; 2 Department of Cancer Genetics, Therapeutics & Ultrastructural Pathology, The Cyprus Institute of Neurology & Genetics, Ayios Dometios, Nicosia 2371, Cyprus

**Keywords:** aldehyde dehydrogenase 3A1, cornea, corneal epithelium, corneal homeostasis, antioxidant, oxidative stress, DNA damage response, DNA damage, cell cycle regulation, wound healing, nuclear localization

## Abstract

Aldehyde dehydrogenase 3A1 (ALDH3A1) oxidizes medium-chain aldehydes to their corresponding carboxylic acids. It is expressed at high rates in the human cornea, where it has been characterized as a multi-functional protein displaying various cytoprotective modes of action. Previous studies identified its association with the DNA damage response (DDR) pathway. Here, we utilized a stable transfected HCE-2 (human corneal epithelium) cell line expressing ALDH3A1, to investigate the molecular mechanisms underlying the cytoprotective role(s) of ALDH3A1. Our data revealed morphological differences among the ALDH3A1-expressing and the mock-transfected HCE-2 cells accompanied by differential expression of *E-cadherin*. Similarly, the ALDH3A1/HCE-2 cells demonstrated higher mobility, reduced proliferation, upregulation of *ZEB1,* and downregulation of *CDK3,* and *p57*. The expression of ALDH3A1 also affected cell cycle progression by inducing the sequestration of HCE-2 cells at the G2/M phase. Following 16 h cell treatments with either H_2_O_2_ or etoposide, a significantly lower percentage of ALDH3A1/HCE-2 cells were apoptotic compared to the respective treated mock/HCE-2 cells. Interestingly, the protective effect of ALDH3A1 expression under these oxidative and genotoxic conditions was accompanied by a reduced formation of γ-H2AX foci and higher levels of total and phospho (Ser15) p53. Finally, ALDH3A1 was found to be localized both in the cytoplasm and the nucleus of transfected HCE-2 cells. Its cellular compartmentalization was not affected by oxidant treatment, while the mechanism by which ALDH3A1 translocates to the nucleus remains unknown. In conclusion, ALDH3A1 protects cells from both apoptosis and DNA damage by interacting with key homeostatic mechanisms associated with cellular morphology, cell cycle, and DDR.

## 1. Introduction

Aldehyde dehydrogenases (ALDHs) comprise a large group of NAD(P)^+^-dependent proteins responsible for the oxidation of aldehydes to their carboxylic acids. Due to their ability to detoxify a wide spectrum of endogenous and exogenous aldehydes, they are considered crucial for cellular antioxidant defense mechanisms [1]. Apart from possessing mere metabolic roles, many ALDH members have been characterized as multifunctional proteins as they are involved in various cellular processes, such as embryogenesis, development, and differentiation [2].

Aldehyde dehydrogenase 3A1 (ALDH3A1) is a homodimeric, cytoplasmic protein, and is a member of the ALDH superfamily. It is constitutively expressed in tissues with high antioxidant needs, such as the stomach, urinary bladder, skin, cornea, and lung, and its expression is inducible in the liver as a response to xenobiotic exposure [3,4,5]. Due to its high expression in the cornea of most mammals (up to 50% of the water-soluble proteins), it has been characterized as a corneal crystallin and it has been attributed with certain structural properties [6,7,8]. ALDH3A1 oxidizes medium-chain aldehydes, such as octanal, hexanal, 4-hydroxy-nonenal, and benzaldehyde [9].

Considering its specificity for toxic aldehydes that are by-products of lipid peroxidation, ALDH3A1 is a crucial component of the antioxidant cellular defense system. It is well established that ALDH3A1 protects corneal epithelial cells from 4-HNE and UV-induced protein oxidation and apoptosis [7,10]. Nevertheless, novel experimental data support that ALDH3A1 exerts its cytoprotective role through additional, non-catalytic functions. For instance, it has been proposed that ALDH3A1 exhibits chaperone-like activity thus protecting corneal epithelial cells from protein aggregation, and consequently preventing the formation of corneal opacities. Quite interestingly, *Aldh3a1* knockout mice presented with a phenotype of corneal haze or clouding, analogous to lens cataracts [11]. Additionally, ALDH3A1 appears to be implicated in cellular functions, such as cell cycle progression, and cellular proliferation [8,12].

The implication of ALDH3A1 in key homeostatic processes has been demonstrated by previous research findings. Specifically, transfected corneal epithelial cells expressing human ALDH3A1 exhibited lower plating efficiency, decreased DNA synthesis, elongated cell cycle, and altered expression/activation of cell cycle regulators [13,14]. Similarly, ALDH3A1 has been associated with the protection of corneal cells against 4-HNE-, UVC-, hydrogen peroxide-, etoposide-, and mitomycin-induced apoptosis [13]. Finally, the expression of ALDH3A1 was downregulated in replicating explant primary cultures of the human corneal epithelium, suggesting an inverse association of ALDH3A1 with proliferation [14].

Previously we established, via stable transfection, an isogenic cell line pair of HCE-2 (Human Corneal Epithelial) cells differing in the expression of ALDH3A1. We demonstrated that HCE-2 cells overexpressing ALDH3A1 exhibited higher viability after hydrogen peroxide and tert-butyl peroxide treatments compared to the mock-transfected HCE-2 cells [12]. Similarly, ALDH3A1 expression resulted in higher resistance against hydrogen peroxide- and tert-butyl peroxide-induced genotoxic and oxidative effects in HCE-2 cells. Finally, the isogenic cell lines exhibited differential expression of a panel of genes related to DNA damage response, apoptosis, cell cycle regulation, DNA damage repair, and ATM/ATR signaling, as illustrated by a pathway-focused RT2 profiler™ PCR array [5]. Based on these observations, in the present study, we sought to further investigate the modes of action underlying the cytoprotective role(s) of ALDH3A1 regarding its association with (i) cell cycle progression and proliferation, (ii) DDR, and (iii) apoptosis in human corneal epithelial cells in an approach to better understand the molecular mechanisms underlying corneal epithelial physiology.

## 2. Results

### 2.1. Morphological Characterization of the Isogenic HCE-2 Cell Line Pair

**Figure 1:**
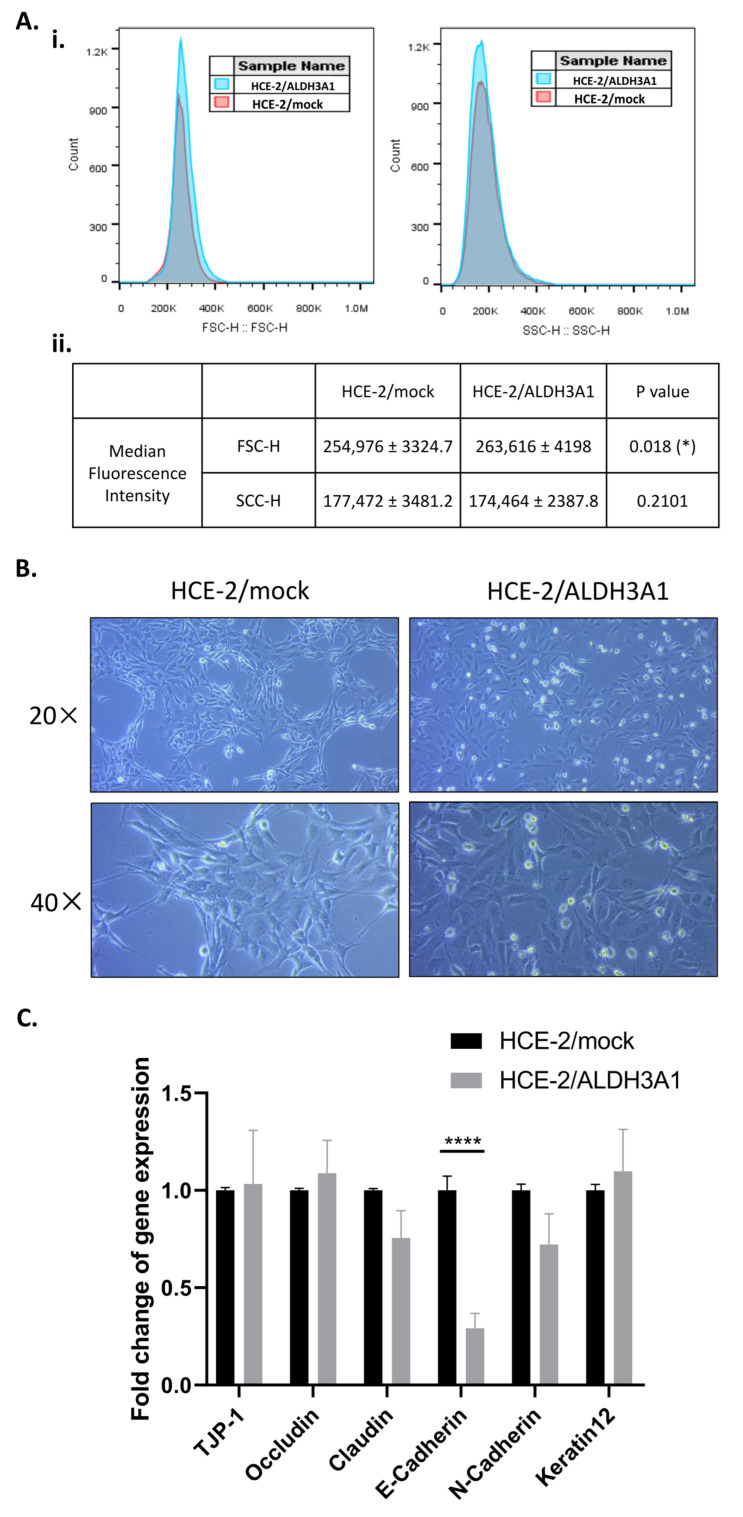
ALDH3A1-expressing and mock-transfected HCE-2 cells exhibit morphological differences. (**A**). The median fluorescence intensity of the forward scatter (FCS) and side scatter of ALDH3A1/HCE-2 and mock/HCE-2 cells were estimated through flow cytometry. At least 2 × 10^4^ events were analyzed per sample. (**i**). Representative histograms of FSC (left) and SSC (right) of the HCE-2 cells. (**ii**). Average values of the FCS and SCS median fluorescence intensity from three independent experiments. The *p*-value represents the statistical significance of the morphological differences among the ALDH3A1/and mock/HCE-2 cell lines. (**B**). Phase contrast microscopy of ALDH3A1/HCE-2 and mock-transfected cells. Figures are representative of 10 random fields for each cell line. (**C**). Comparative transcriptional expression levels of *TJP-1*, *occludin*, *claudin*, *E-cadherin*, *N-cadherin*, and *keratin 12* among the isogenic HCE-2 cell lines. The expression levels were normalized to the respective β-actin levels. Mock-transfected HCE-2 cells were used as the reference sample. The ΔΔCt method was used for data quantification. The results are shown as mean ± S.E. (**A**,**C**). Table and graph are representative of at least three independent experiments. * *p* ≤ 0.05, **** *p* < 0.0001.

An isogenic human corneal epithelial cell line (HCE-2) stably expressing ALDH3A1 was previously established by our group [12]. In this study, we were prompted to investigate the morphology of the ALDH3A1-expressing HCE-2 cells (HCE-2/ALDH3A1) compared to the mock-transfected HCE-2 cells (HCE-2/mock). To this end, we utilized flow cytometry to determine the median fluorescence intensity of the forward (FSC-H) and side (SCC-H) scatter of the two samples (Figure 1A) [15].

Our analysis indicated only a small, however statistically significant, increase in the size of the ALDH3A1-expressing HCE-2 cells compared to the mock-transfected cells (Median Fluorescence Intensity-MFI of FSC-H, Figure 1A). No difference was observed in the internal complexity of the isogenic HCE-2 cell lines through this method (MFI of SSC-H, Figure 1A). Additionally, optical observation of the isogenic cell lines revealed strong culture morphological differences, with HCE-2/ALDH3A1 cells exhibiting lower cellular density and weaker cell–cell adhesion (Figure 1B) compared to the HCE-2/mock cells. Therefore, we investigated, through qPCR, the expression of a set of genes associated with cellular morphology and cell–cell interactions. Our data revealed that *E-cadherin* was significantly downregulated (0.29-fold change, *p* < 0.0001) in the ALDH3A1-expressing cells in comparison to the mock/HCE-2 cells (Figure 1C). *N-cadherin* exhibited only a small, non-significant downregulation (0.72-fold change, *p* ≤ 0.05). No significant differentiation was observed at the expression levels of the other examined genes (Figure 1C).

### 2.2. ALDH3A1 Expression Promotes the Wound-Healing Potential of HCE-2 Cells

**Figure 2:**
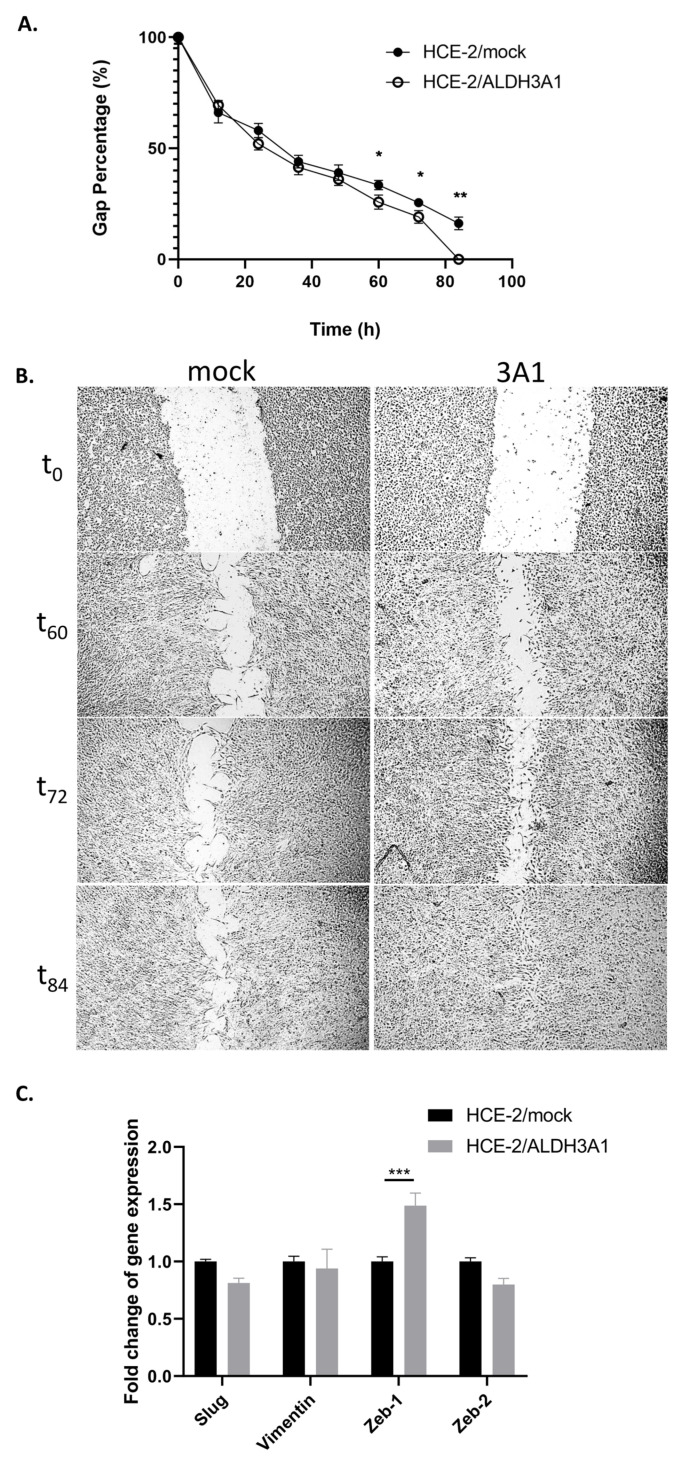
ALDH3A1-expressing HCE-2 cells exhibit increased wound-healing capacity compared to the mock/HCE-2 cells. (**A**,**B**). Scratch assay: cellular expansion and “wound” closure were monitored through an optical microscope (magnification 20×) at various time points. (**A**). Quantification of wound closure as the residual gap percentage by Image J analysis. (**B**). Representative photos by phase contrast microscopy (20×) at 0, 60, 72, and 84 h past the scratch introduction. (**C**). Comparative analysis of the mRNA levels of *slug*, *vimentin*, *ZEB1*, and *ZEB2* through comparative real-time PCR. The expression levels were normalized to that of *β-actin*. Mock/HCE-2 cells were used as the reference sample. The ΔΔCt method was used for data quantification. (**A**,**C**). Graphs are representative of at least three independent experiments. The results are shown as mean ± S.E. * *p* ≤ 0.05, ** *p* < 0.01, *** *p* < 0.001.

Considering the significant downregulation of *E-cadherin*, as well as the differentiated two-dimensional expansion of the ALDH3A1-expressing HCE-2 cells, we employed the wound-healing assay to explore the ability of cells to “spread” and cover a scratch introduced by a pipette tip. Our data showed that the ALDH3A1/HCE-2 cells exhibited a higher wound-healing potential than the mock-transfected HCE-2 cells, as illustrated by the % percentage of gap closure at the indicated time points (t_0_–t_84_) (Figure 2A,B). Specifically, ALDH3A1/HCE-2 cells managed to close the introduced gap after roughly 84 h, while at the same time, 16.83% of the initial gap was still open in the mock/HCE-2 cells (Figure 2A,B).

Therefore, we analyzed through QPCR analysis the expression of several wound-healing markers. Our data indicated that ALDH3A1 was associated with higher mRNA levels of *ZEB1* (1.49-fold change, *p* < 0.001) (Figure 2C). On the contrary, no difference was observed among the two isogenic cell lines in the case of *slug*, *vimentin*, and *ZEB2* (Figure 2C).

### 2.3. ALDH3A1 Is Associated with Slower Proliferation Rate and a G2/M Delay in HCE-2 Cells

**Figure 3:**
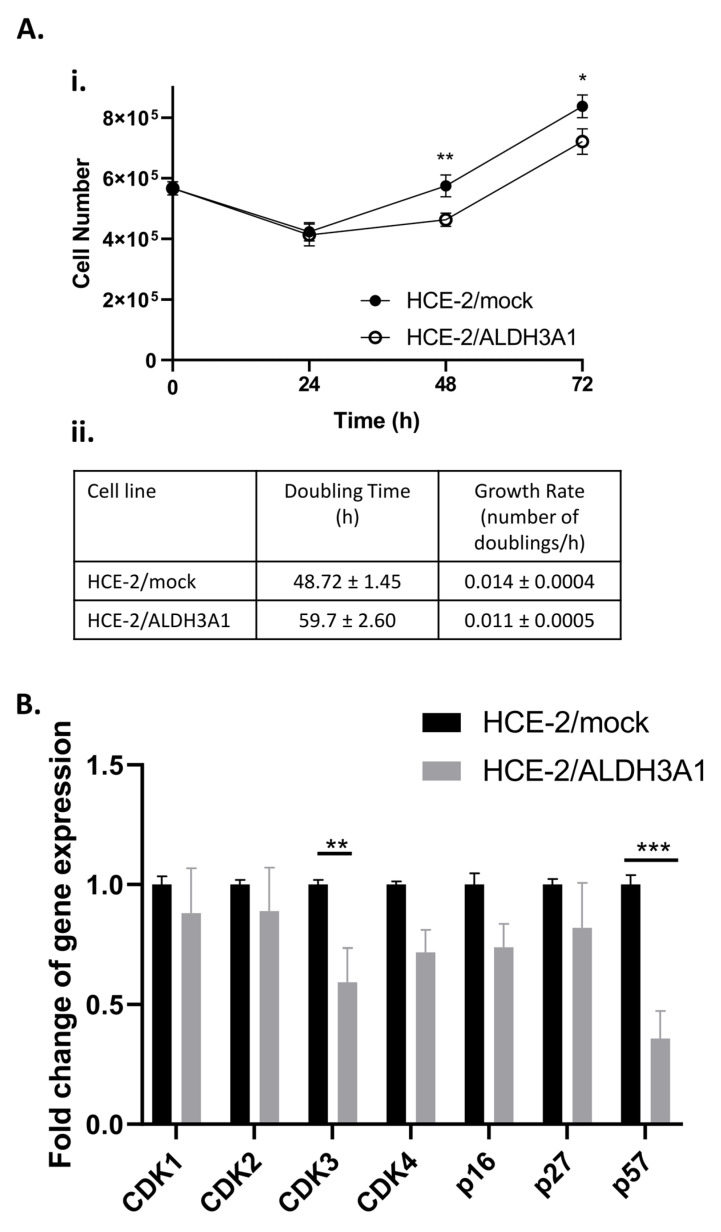
ALDH3A1/HCE-2 cells demonstrate a slower growth rate compared to mock/HCE-2 cells. (**A**). Proliferation of the isogenic HCE-2 cell lines. (**i**). HCE-2/mock and HCE-2/ALDH3A1 cells (4 × 10^5^) were seeded in 10 cm culture plates and cell numbers were determined every 24 h for 72 h (**ii**). Data were used to calculate the growth rate (GR) and doubling time (DT) of the isogenic cell lines (**B**). Fold-change of expression of *CDK1*, *CDK2*, *CDK3*, *CDK4*, *p16*, *p27*, and *p57* though quantitative real-time PCR. The mRNA levels were normalized to that of *β-actin*. Mock-transfected HCE-2 cells were used as a reference sample. The ΔΔCt method was used for data quantification. (**A**,**B**). The results are shown as mean ± S.E. The graphs are representative of at least three independent experiments. * *p* ≤ 0.05, ** *p* < 0.01, **** *p* < 0.0001.

The results from the wound-healing analysis, in combination with previous studies indicating the negative effect of ALDH3A1 expression on cellular growth, prompted us to investigate the proliferation potential of the HCE-2 isogenic cell lines. Our findings demonstrated that the ALDH3A1-expressing cells exhibited significantly slower proliferation rates compared to the mock-transfected HCE-2 cells (Figure 3(Ai)).

The doubling time (DT) of ALDH3A1/HCE-2 cells (59.7 h) was higher than the DT of mock/HCE-2 cells (48.72 h), while the growth rate (GR) of the ALDH3A1-expressing cells (0.011 doublings/h), was lower than the respective GR of the mock-transfected HCE-2 cells (0.014 doublings/h) (Figure 3(Aii)). To further investigate the effect of ALDH3A1 on HCE-2 proliferation, we comparatively examined the mRNA levels of certain cell-cycle-related genes in the ALDH3A1/HCE-2 and mock/HCE-2 cells. Our results demonstrated that ALDH3A1 expression resulted in a significant downregulation of *CDK3* (0.59-fold change, *p* < 0.01) and *p57* (0.36-fold change, *p* ≤ 0.001). Only negligible differentiations in the mRNA levels of *CDK1*, *CDK2*, *CDK4*, and *p16* were observed between the isogenic HCE-2 cell lines (Figure 3B).

**Figure 4:**
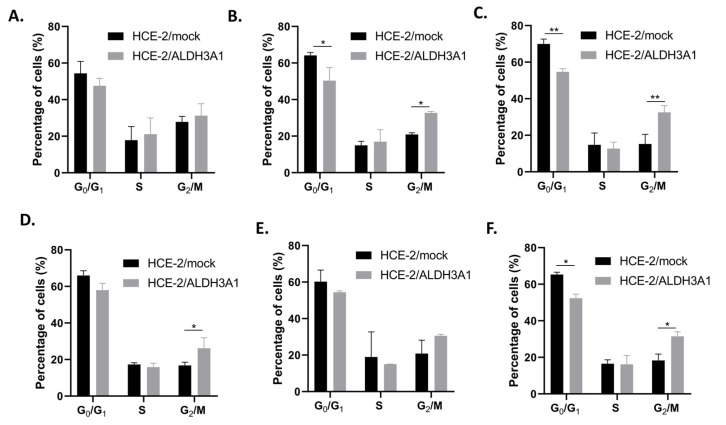
ALDH3A1 expression induces a G2/M delay in HCE-2 cells. (**A**–**F**). Cell cycle kinetics of the ALDH3A1-expressing and mock-transfected HCE-2 cells. The cell cycle of asynchronized HCE-2 cells was analyzed by flow cytometry at (**A**) 0, (**B**) 16, (**C**) 24, (**D**) 36, (**E**) 48, and (**F**) 72 h. A minimum of 20,000 events were analyzed per sample. The graphs are representative of three independent experiments. The results are shown as mean ± S.E. * *p* ≤ 0.05, ** *p* < 0.01.

Due to the differentiated expression of certain key cell cycle regulators in the ALDH3A1/HCE-2 cells, we examined the progression of the cell cycle in the isogenic HCE-2 cell lines. Our flow cytometric analysis revealed that ALDH3A1-expressing HCE-2 cells exhibited a G2/M phase delay, considering that a higher percentage of ALDH3A1/HCE-2 cells were at the G2/M phase in comparison to the mock/HCE-2 cells at all the examined time points (Figure 4A–F).

More specifically, 27.84% and 31.22% of mock/HCE-2 and ALDH3A1/HCE-2 cells, respectively, were in the G2/M phase at t_0_, while the percentages of cells in the G2/M phase were 32.57% and 31.49% for ALDH3A1/HCE-2 vs. 15.26% and 18.28% for mock/HCE-2 at 24 h and 72 h, respectively (Figure 4A,C,F).

### 2.4. ALDH3A1 Protects HCE-2 Cells from the Cytotoxic Effects of H_2_O_2_ and Etoposide by Exhibiting Anti-Apoptotic Properties

**Figure 5:**
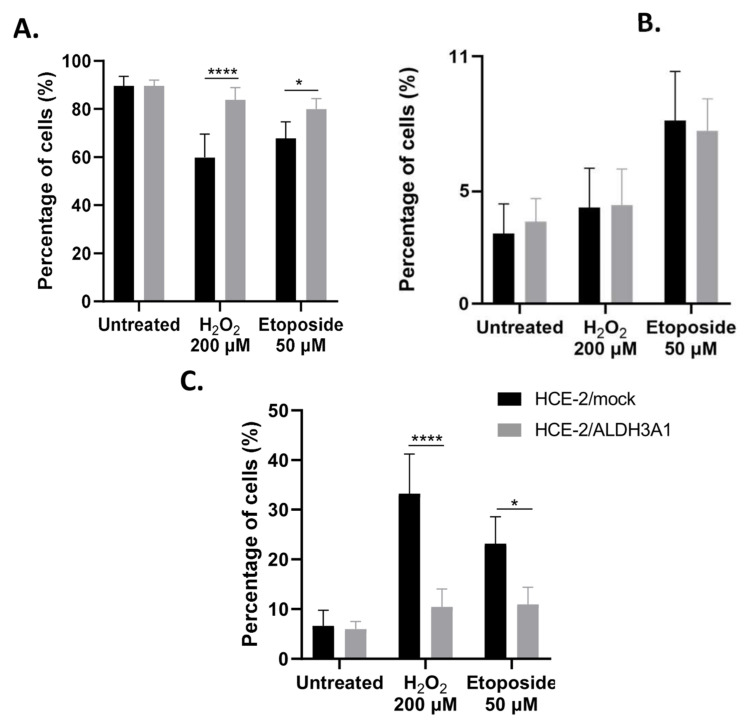
ALDH3A1 expression protects HCE-2 cells from H_2_O_2_- and etoposide-induced apoptosis. (**A**–**C**). Flow cytometry analysis of apoptosis in the ALDH3A1-expressing and mock-transfected HCE-2 cells. The isogenic cell lines were either left untreated or treated with 200 μM H_2_O_2_ or 50 μM etoposide for 16 h. Then, propidium iodide and annexin-V FITC staining was used to analyze the proportion of (**A**) live, (**B**) early apoptotic, and (**C**) late apoptotic ALDH3A1/HCE-2 and mock/HCE-2 cells. A minimum of 20,000 events were analyzed per sample. The graphs are representative of three independent experiments. * *p* ≤ 0.05, **** *p* < 0.0001.

Previous studies have demonstrated the ability of ALDH3A1 to protect HCE-2 cells from the genotoxic and cytotoxic effects of etoposide and H_2_O_2_ [5,12]. Therefore, in this study, we investigated the H_2_O_2_- and etoposide-induced apoptosis of the isogenic cell line pair. ALDH3A1/HCE-2 and mock/HCE-2 cells were treated with either 200 μM H_2_O_2_ or 50 μM etoposide for 16 h and then, the percentages of living, early apoptotic, and late apoptotic cells were determined via flow cytometry analysis. Our data indicated that the ALDH3A1-expressing cells exhibited much higher viability compared to the mock-transfected cells after treatments with both agents. Specifically, 59.9% and 67.86% of mock/HCE-2 cells were viable under treatments with H_2_O_2_ and etoposide, respectively, while the percentages were 83.9% and 79.96% for the ALDH3A1-expressing cells under the same treatment conditions (Figure 5A).

No significant difference was observed in the percentage of early apoptotic cells between the isogenic HCE-2 cell lines (Figure 5B), while a significantly higher percentage of H_2_O_2_- and etoposide-treated mock/HCE-2 cells were late apoptotic compared to the respectively treated ALDH3A1/HCE-2 cells (Figure 5C). Specifically, the percentage of late apoptotic H_2_O_2_-treated mock/HCE-2 cells (33.22%) was approximately three-fold higher than the respective percentage of the H_2_O_2_ -treated ALDH3A1/HCE-2 cells (10.44%) (Figure 5C). Similarly, 23.13% of the etoposide-treated mock/HCE-2 were late apoptotic, which was approximately double the percentage of the late apoptotic cells observed for the etoposide-treated ALDH3A1/HCE-2 cells (10.95%) (Figure 5C).

### 2.5. The Cytoprotective Effect of ALDH3A1 Is Accompanied by Altered Expression and/or Activation of H2AX and p53

**Figure 6:**
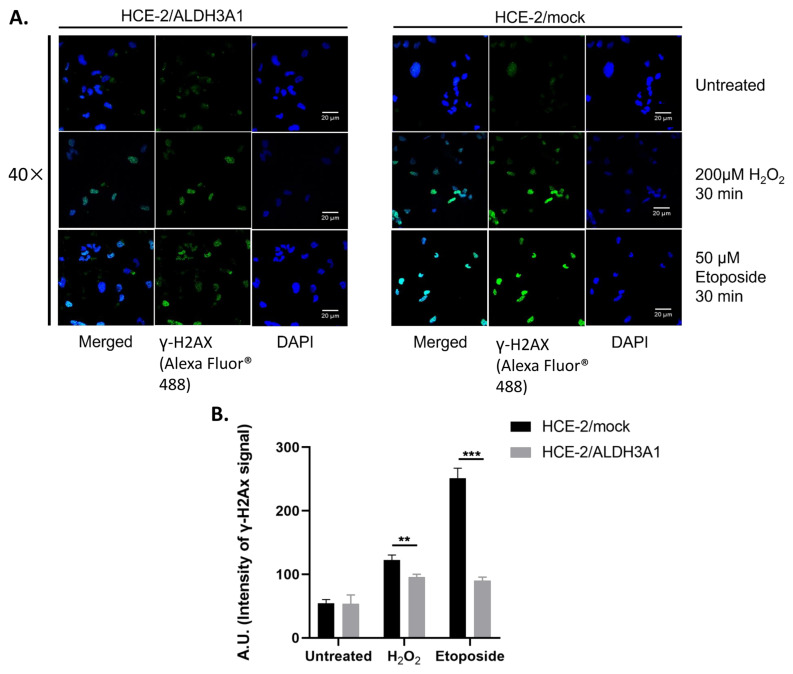
ALDH3A1 expression is associated with reduced γ-H2AX foci formation under oxidative and genotoxic conditions. The formation of gamma-H2AΧ foci in the ALDH3A1/HCE-2 and mock/HCE-2 cells under the influence of 200 μM H_2_O_2_ and 50 μM etoposide (30 min) was determined through immunofluorescence for γH2AX (green). Nuclei were stained with DAPI (4′,6-diamino-2-phenylindole) (blue). (**A**) Cells were visualized (magnification 40×) with Andor Ixon + 885 digital camera on a customized Andor Revolution Spinning Disk Confocal System built around an IX81; Olympus stand (CIBIT Facility, MBG-DUTH). Figures are representative of 10 random fields for each examined condition. Scale bar denotes 20 μm. (**B**) The levels of γH2AX foci were quantified (in Arbitrary Units, AU) by classifying 100 cells from random fields for each sample and summing their scores. The results are shown as mean ± S.E. At least three independent experiments were performed for each condition tested. ** *p* < 0.01, *** *p* < 0.001.

The effect of ALDH3A1 on cell cycle progression, proliferation, and apoptosis prompted us to examine the expression and/or activation of H2AX and p53 under oxidative and genotoxic conditions. For this reason, the isogenic HCE-2 cell lines were treated with H_2_O_2_ (200 μM) or etoposide (50 μM) for 30 min, and then, the activation of H2AX was detected by visualizing the formation of the phospho-γH2AX (Ser-139) foci in the nucleus of HCE-2 cells through immunofluorescence (Figure 6A).

Our results indicated that the ALDH3A1/HCE-2 cells exhibit significantly lower levels of γH2AX foci formation compared to the mock-transfected HCE-2 cells under all conditions tested (Figure 6A,B). The higher differentiation was observed after treatments with etoposide, where the arbitrary units of γ-H2AX signal intensity of the mock/HCE-2 cells were approximately three-fold higher than the respective levels of the ALDH3A1-expressing HCE-2 cells (Figure 6B).

**Figure 7:**
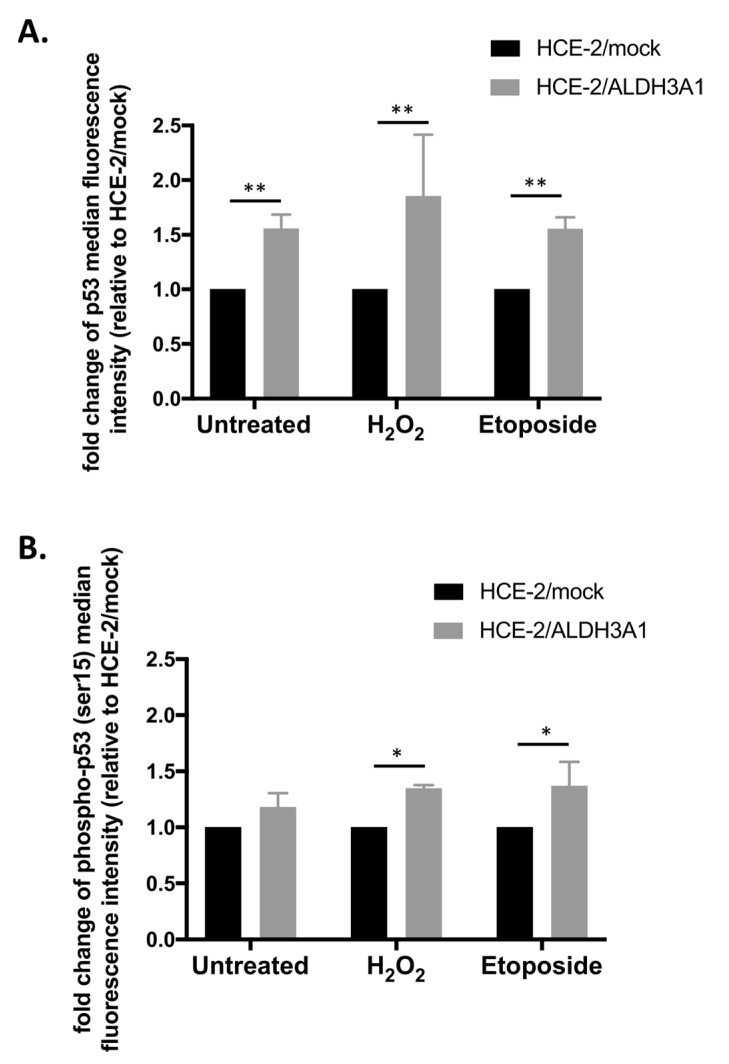
ALDH3A1 expression is associated with higher levels of total and phosphorylated (Ser15) p53. Flow cytometry analysis was utilized for analyzing the levels of (**A**) total and (**B**) phospho (Ser15) p53 in untreated as well as in treated with either H_2_O_2_ (200 μM) or etoposide (50 μM) (24 h) ALDH3A1/HCE-2 and mock/HCE-2 cells. A minimum of 10,000 events were analyzed per sample. The graphs are representative of three independent experiments. * *p* ≤ 0.05, ** *p* < 0.01.

Next, the isogenic cell line pair was treated with 200 μM H_2_O_2_ or 50 μM etoposide for 24 h min, and the total p53 protein levels as well as the phosphorylation (Ser15) levels of p53 were investigated through flow cytometry analysis (Figure 7).

Our analysis revealed that the ALDH3A1-expressing cells exhibited significantly higher levels of total p53, both in untreated cells as well as under the influence of either H_2_O_2_ or etoposide (Figure 7A). Similarly, we demonstrated higher phosphorylation of p53 in the ALDH3A1/HCE-2 cells compared to mock/HCE-2 cells after the H_2_O_2_ and etoposide treatments (Figure 7B). No significant alterations in the phosphor-p53 levels were observed among the untreated ALDH3A1-expressing and mock-transfected HCE-2 cells (Figure 7B).

### 2.6. ALDH3A1 Is Localized in the Nucleus of HCE-2 Cells through an Unclarified Mechanism

**Figure 8:**
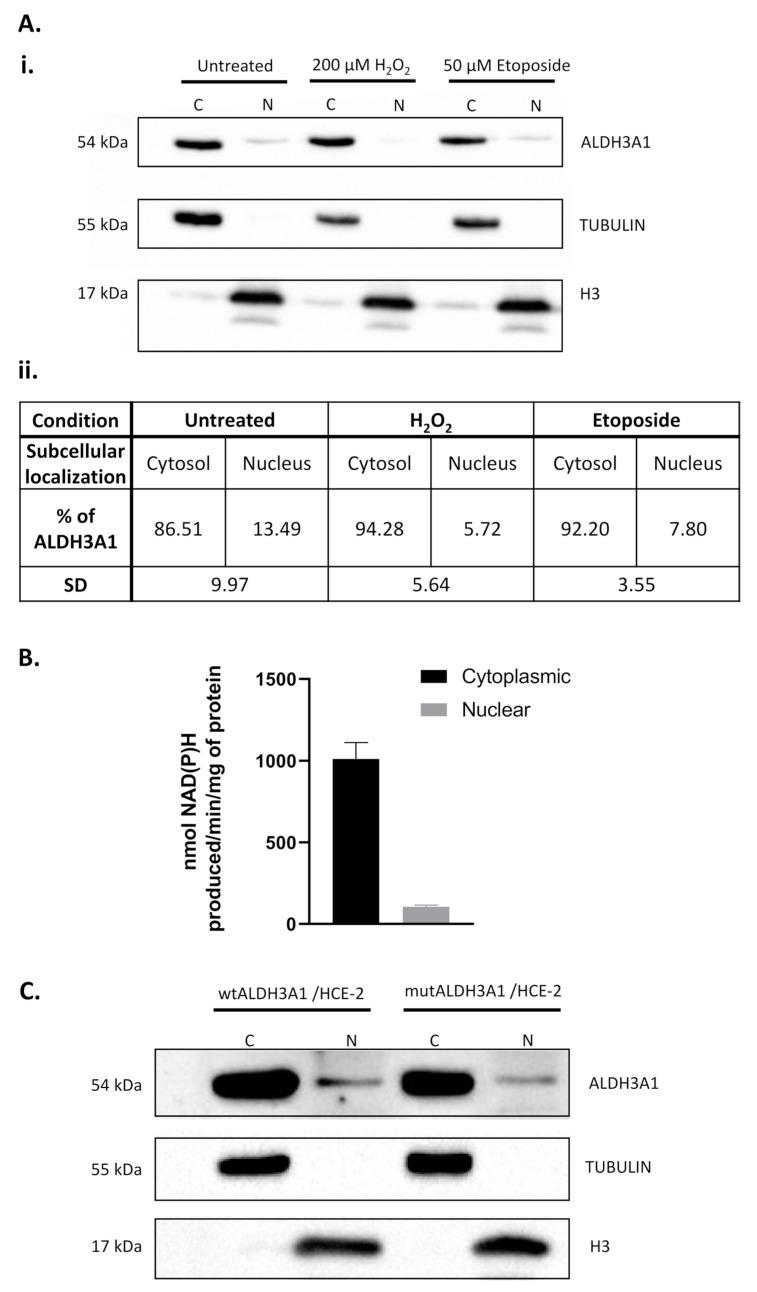
ALDH3A1 is located both in the cytoplasm and nucleus of HCE-2 cells and oxidative stress conditions do not alter its translocation. (**Ai**). The localization of ALDH3A1 was determined by immunoblotting. ALDH3A1/HCE-2 cells were left untreated or treated with either 200 mΜ H_2_O_2_ or 50 μM etoposide for 16 h and then their nuclear and cytoplasmic extracts were examined by Western blot analysis. (**Aii**). Quantitation of the signal intensity of ALDH3A1 antibody at the cytoplasmic and nuclear extracts expressed respectively as a percentage of total signal intensity. (**B**). Determination of the enzymatic activity of ALDH3A1 using benzaldehyde as a substrate in the cytoplasmic and nuclear extracts of the untreated ALDH3A1/HCE-2 cells. (**C**). The localization of the wild-type and NLS-mutated ALDH3A1 was identified in HCE-2 cells by Western blotting. HCE-2 cells were transiently transfected with either wild-type or NLS-mutated hALDH3A1 constructs. Transfected cells were then lysed, and the nuclear and cytoplasmic extracts were prepared and immunoblotted against ALDH3A1. (**A**,**C**). Tubulin and histone H3 were used as internal controls for the purity of the nuclear and cytoplasmic extracts. Figures are representative of three independent experiments.

Our experimental findings indicated the implication of ALDH3A1 in the expression and/or activation of certain proteins located at the nucleus. Here, we studied the localization of ALDH3A1 in HCE-2 cells under both “physiological” and stress conditions. For this reason, we treated ALDH3A1/HCE-2 cells with 200 μM H_2_O_2_ or 50 μM etoposide for 16 h, and then their nuclear and cytosolic extracts were subjected to immunoblotting (Figure 8A).

Our data demonstrated the existence of ALDH3A1 mainly in the cytosolic extracts, but also at a smaller percentage, in the nuclear cell extracts under all conditions tested (Figure 8A). However, treatment with either H_2_O_2_ or etoposide did not further enhance the nuclear localization of ALDH3A1 (Figure 8A). The subcellular localization of ALDH3A1 was also validated by measuring its enzymatic activity in the nuclear and cytoplasmic extracts of the ALDH3A1/HCE cells (Figure 8B).

Next, we explored the mechanism underlying the transportation of ALDH3A1 from the cytosol to the nucleus. A previous study by Pappa et al. (2005) reported the existence of a potential bipartite nuclear localization signal (NLS) at residues 265–281 of the protein’s amino acid sequence (265KKSLKEFYGEDAKKSRD281) [14]. In this study, we validated this putative NLS signal by performing site-directed mutagenesis and consequently substituting lysines 265, 266, 277, and 278 with alanines on the hALDH3A1 plasmid, that was constructed and described previously [16]. Then, HCE-2 cells were transiently transfected with both wild-type hALDH3A1 (wtALDH3A1/HCE-2) and mutated for the potential NLS signal (mutALDH3A1/HCE-2) constructs and the cellular localization of ALDH3A1 was detected by Western blotting. Our results demonstrated that both wild-type (wtALDH3A1) and mutated (mutALDH3A1) ALDH3A1 were found at the nucleus of HCE-2 cells, indicating that the sequence identified as the potential NLS could not possibly be implicated in the nuclear translocation of ALDH3A1 (Figure 8C).

## 3. Discussion

ALDH3A1 is a multifaceted enzyme, expressed in tissues with increased antioxidant needs. In the cornea, it exhibits, apart from its metabolic role, other functions associated with the cell growth and optical properties of the tissue. In a previous report, our group demonstrated the protective properties of ALDH3A1 under stress conditions and its effect on the regulation of genes involved in the DNA damage response (e.g., ATM/ATR signaling, and DNA damage repair) [5]. In this study, we focused on the unclarified cytoprotective properties of ALDH3A1 in human corneal epithelium. For this reason, we utilized a previously established isogenic HCE-2 (human corneal epithelial) cell line pair differing only in the expression of ALDH3A1 [5,12]. Our findings demonstrated that ALDH3A1 expression was associated with a slower proliferation rate, higher mobility, morphological alterations, and differential expression of *E-cadherin*, *ZEB1*, *CDK3*, and *p57*. Additionally, flow cytometry analysis of the cell cycle revealed an accumulation of ALDH3A1/HCE-2 cells in the G2/M phase. Treatments of the isogenic HCE-2 cells revealed that the ALDH3A1 expression protected HCE-2 cells from H_2_O_2_- and etoposide-induced apoptosis. The anti-apoptotic and cell-cycle-altering effects of ALDH3A1 were accompanied by the altered expression/regulation of γ-H2AΧ and p53. Finally, ALDH3A1 was found to be localized in the nucleus, and treatment with oxidative agents (H_2_O_2_ or etoposide) did not alter its cellular compartmentalization. The potential NLS signal of ALDH3A1 that was previously reported (a bipartite NLS signal) [14] was further validated in this study and found not to be responsible for the nuclear translocation of the protein.

Our experimental data showed that the ALDH3A1-expressing HCE-2 cells exhibited certain differentiations in their morphological characteristics, including a small change in their size and a less-dense cellular culture. This phenotype was accompanied by a significant downregulation of *E-cadherin.* E-Cadherin is a calcium-dependent protein involved in the formation of adherent junctions among neighboring cells [17]. Apart from its role in cell–cell adhesion, it is considered a crucial factor for the maintenance of the epithelial phenotype of cells as well as for contact inhibition [18]. Consequently, downregulation of E-cadherin is associated with higher cellular mobility and metastasis in cancer [19]. The downregulation of *E-cadherin* as a result of ALDH3A1 expression is in line with our optical observations demonstrating the formation of denser cultures with more adhered cells in the case of the non-expressing, compared to the ALDH3A1-expressing, HCE-2 cells. To the extent of our knowledge, this is the first time that the cell morphology and the expression of *E-cadherin* in relation to ALDH3A1 are being studied in corneal epithelial cells. However, the association of ALDH3A1 with E-cadherin expression has been previously studied in cancer cells. Specifically, Terzuoli et al. (2019) demonstrated that ALDH3A1^high^ non-small-cell lung carcinoma (NSCLC) and melanoma cells exhibited downregulation of E-cadherin compared to ALDH3A1^low^ cells [20]. Similarly, Wu et al. (2016) reported that ALDH^bright^ SGC-7901 (gastric cancer cell lines) cells had lower mRNA levels of E-cadherin compared to ALDH^low^ SGC-7901 cells [21].

Next, we examined the wound-healing potential of ALDH3A1 and showed that the ALDH3A1/HCE-2 cells have higher mobility/wound-healing capacity compared to the mock-transfected HCE-2 cells. We also examined, through QPCR, the expression rates of *slug*, *vimentin*, *ZEB1*, and *ZEB2,* and showed that *ZEB1* is significantly upregulated in the ALDH3A1-expressing HCE-2 cells. ZEB1 (Zinc finger E-box binding homeobox 1) is a transcription factor, known for its role in the Epithelial–Mesenchymal Transition (EMT) in cancer. Specifically, overexpression of ZEB1 is associated with EMT, tumor invasion, and metastasis [22]. In non-cancerous epithelial cells, ZEB1 attenuates cell adhesion, partly by downregulating E-cadherin and other cell-adhesion-related proteins [23,24]. Consequently, it promotes mobility and de-differentiation of epithelial cells [24]. Feng et. al. (2004) examined the expression and enzymatic activity of ALDH3A1 after an alkali burn in mice cornea. They demonstrated that ALDH3A1 is downregulated as a result of alkali burn, however, its expression is gradually restored during healing [25]. Additionally, Terzuoli et. al. (2019) showed that ALDH3A1 expression correlated with an increased expression of ZEB1 in HCC4006 (metastatic NSCLC) cells [20]. To this end, our observations suggest that ALDH3A1 expression induces the transition of HCE-2 cells from a phenotype with tight cell–cell interactions towards a state with low cell–cell adhesion with high regenerative properties.

Subsequently, we demonstrated that ALDH3A1 had a negative effect on the cellular proliferation of HCE-2 and induced the downregulation of *CDK3*, and *p57*. Cyclin-dependent kinases (CDKs) comprise a subfamily of serine/threonine kinase proteins involved in the control of cell cycle progression and proliferation. CDKs activity is positively regulated through the formation of heterodimeric complexes with specific cyclins [26]. This complex is responsible for modulating the progression of cell cycle phases. Protein p57, encoded by gene *CDKN1C*, is a cyclin/CDK inhibitor that causes the arrest of cells at the G1 phase [27]. In addition, p57 is involved in the differentiation of certain cell types both through CDKs inhibition and thus cell cycle exit, as well as through mechanisms that are not related to CDKs [28]. The differential expression of these cell cycle regulators does not come as a surprise considering the effect of ALDH3A1 on cell growth. Our data are in line with a previous study by Pappa et al. (2005) showing that ALDH3A1 exhibits suppressive functions on cell proliferation accompanied by reduced CDK activity and altered protein levels of cyclins A, B, and E, E2F1 and p21 in HCE (human corneal epithelial) cells [14]. Additionally, Qu et al. (2020) demonstrated that the stable transfection of SCC15 and SCC25 (oral squamous carcinoma cell lines) cells to express ALDH3A1 resulted in slower proliferation rates. However, in contrast to our study, they showed that the ALDH3A1-expressing SCC15 and SCC25 cells also exhibited weaker migration and invasion potential [29].

Our results on the effect of ALDH3A1 on cell proliferation prompted us to study the progression of cell cycle on the isogenic cell line pair. Our flow cytometry analysis revealed a “delay” in ALDH3A1/HCE-2 cells at the G2/M phase. To the extent of our knowledge, this is the first time that the effect of ALDH3A1 on cell cycle progression is being studied in human corneal cells. Qu et al. (2020) performed a similar study, however in oral squamous carcinoma cells, and reported that, in agreement with our results, the ALDH3A1-expressing SCC15 and SCC25 cells also exhibited a delay at the G2/M phase [29].

Next, we treated HCE-2 cells with either H_2_O_2_ or etoposide for 16 h and investigated their apoptotic status by flow cytometry. We chose these specific stressors due to their different mode of action. H_2_O_2_ is a common oxidative agent, while etoposide acts mainly through inducing double-strand DNA break formation by inhibiting topoisomerase II [30,31]. Our data revealed that ALDH3A1 expression exerted an anti-apoptotic role in HCE-2 cells under both conditions tested. Our findings agree with the findings reported in previous studies. Specifically, Saiki et al. (2018) reported that a higher percentage (2.2 fold) of salivary stem/progenitor cells (SSPCs) isolated from irradiated *Aldh3a1*(-/-) murine submandibular glands (SMGs) were early or late apoptotic compared to the respective SSPCs isolated from wild-type SMGs [32]. Similarly, the anti-apoptotic effect of ALDH3A1 was demonstrated in studies reporting that ALDH3A1 expression protected HCE cells from mitomycin C, VP-16, and H_2_O_2_-induced DNA fragmentation [7,33]. Along these lines, our group has previously shown that, after a 16 h treatment with either H_2_O_2_ or tert-butyl hydroperoxide, the ALDH3A1-expressing HCE-2 cells managed to retain their viability more effectively than the mock-transfected cells [12].

Next, we observed, through either immunofluorescence or flow cytometry, that the expression of ALDH3A1 is associated with lower activation/foci formation of γH2AX as well as higher protein levels of p53 and phospho-p53(ser15) after treatments with H_2_O_2_ or etoposide. H2AX is being phosphorylated at serine 139 (γ-H2AX) and thus being activated as a response to DNA damage and specifically double-strand breaks [34]. The activation of γ-H2AX subsequently induces its accumulation at the DSBs sites leading to the formation of foci, which are visible through immunofluorescence microscopy. γH2AX is involved in the DNA damage response pathway by recruiting certain DNA repair proteins and it is considered an important, early DNA damage marker [35]. Our findings are in accordance with previously published data, demonstrating lower H_2_O_2_^−^, tert-butyl peroxide-, and etoposide-induced DNA damage levels in ALDH3A1/HCE-2 cells compared to mock-transfected HCE-2 cells [5]. Similarly, Jang et al. (2013) reported that ALDH3A1-overexpressing human bronchial epithelial cells exhibited lower cigarette-smoking-induced H2AX Ser-319 phosphorylation levels [36].

The tumor protein p53 plays a crucial role in the response of cells under different stress conditions, by regulating cell-cycle progression and thus inducing growth arrest or apoptosis [37]. p53 is involved in both antioxidant and DNA damage responses, while it promotes DNA damage repair [38]. Specifically, DNA damage induces the stabilization/activation of p53 through phosphorylation at various sites, with Ser15 being a common phosphorylation target under this specific condition [37]. Subsequently, the activation of p53 results in the induction of a panel of cellular signaling pathways related to DNA damage repair, cell death as well as cell cycle arrest, primarily at the G1/S, but also at the G2/M checkpoint [39]. Indeed, the upregulation of p53 in the ALDH3A1-expressing untreated HCE-2 cells could be associated with the accumulation of the ALDH3A1/HCE-2 cells at the G2/M phase. The increased levels of p53 even under non-stress conditions could be beneficial by maintaining cells in a state of “alertness”. This would enable them to react faster in case of DNA damage, and thus, more effectively induce the DNA damage response. The enhancement of DDR would eventually lead to quicker and more efficient DNA damage repair and increased cell viability. Our data, demonstrating higher levels of total and phospho-p53 along with lower DNA damage and apoptosis levels in the ALDH3A1/HCE-2 cells, are in agreement with this hypothesis. In accordance with our findings, Fan et al. (2021) reported that ALDH3A1 was positively associated with the total p53 in tissues of lung adenocarcinoma and that a knockdown of ALDH3A1 in the lung adenocarcinoma cell line A549 resulted in lower protein levels of total p53 [40]. Similarly, Koppaka et al. (2016), demonstrated that expression of ALDH3A1 in the corneal epithelium hTCEpi cell line was associated with increased cytosolic and nuclear p53 levels. Furthermore, even though knockout of *aldh3a1* did not have any effect on p53, dual knockout of both *aldh3a1* and *aldh1a1* in mice resulted in the total loss of total p53 and phospho-p53 in the corneal tissue [41].

Finally, we studied the sub-cellular localization of ALDH3A1 and found that it is located mainly in the cytoplasmic compartment, but also, to a lesser extent, in the nucleus of the HCE-2 cells under non-stress conditions, and that treatments with either oxidative or genotoxic agents did not further enhance its nuclear localization. The nuclear localization of ALDH3A1 in human corneal epithelial cells has been reported in other studies [14,42]. Interestingly, Pappa et al. (2005) reported a potential bipartite (BP) NLS at the ALDH3A1 amino acid sequence [14]. BP NLS consists of two basic clusters comprised of 2–3 amino acids and a 9–10 amino acid spacer that links these two positively charged regions [43]. In the case of ALDH3A1, this putative NLS is comprised of two lysine regions linked by a 10-residue spacer (265KKSLKEFYGEDAKKSRD281). Therefore, to deactivate this putative NLS, we substituted its basic lysine residues with the non-polar amino acid alanine. Our experiments demonstrated that this substitution did not inhibit the translocation of ALDH3A1 at the nucleus of HCE-2, thus the mechanism underlying ALDH3A1 nuclear localization is not related to this specific sequence. The detection of the protein in the nucleus of corneal cells, implies novel, yet-uncharacterized ALDH3A1 functions, which warrants further investigation. Consequently, the existence of ALDH3A1 in the nucleus of cells could allow its direct interaction with certain regulatory proteins. This mode of action should be unsurprising in relation to the corneal epithelium. For instance, ferritin, an iron-sequestering molecule, mainly known as a metabolic, cytoplasmic protein, was also found to be nuclear in the avian corneal epithelium and the mechanistic studies for its nuclear localization identified a tissue-specific nuclear transporter termed ferritoid. In the nucleus, ferritin was found to directly interact both with DNA and gene regulatory proteins and to exert a protective role against UV-induced oxidative damage [44,45].

## 4. Materials and Methods

### 4.1. Materials

The human corneal epithelium cell line HCE-2 was obtained from ATCC (Manassas, VA, USA). Culture medium, antibiotics, fetal bovine serum (FBS), trypsin, and medium additives were either from Biosera (East Sussex, UK), Gibco (Life Technologies, Carlsbad, CA, USA) or Sigma-Aldrich Co. (St. Louis, MO, USA). Lipofectamine 2000 was purchased from Invitrogen (ThermoFischer Scientific, Waltham, MA, USA) and hygromycin was obtained from Sigma-Aldrich Co. (St. Louis, MO, USA). Etoposide and hydrogen peroxide was purchased by Sigma-Aldrich (St. Louis, MO, USA). The Pierce™ BCA Protein assay was from Thermo Scientific (Rockford, IL, USA). Chemiluminescence reagents and polyvinylidene difluoride (PVDF) membranes were purchased from Millipore (Bedford, MA, USA). The primary antibodies anti-γH2AX, anti-p53, anti-phospho-p53 (ser15), and anti-H3 were from Cell Signaling Technology (Boston, MA, USA), while anti-β tubulin was purchased from Sigma-Aldrich Co. (St. Louis, MO, USA) and anti-ALDH3A1 from Abnova (Taipei City, Taiwan). Goat anti-mouse and anti-rabbit IgG horseradish-peroxidase-conjugated antibodies were purchased from Millipore (Bedford, MA, USA), goat anti-rabbit Alexa Fluor^®^ 488-conjugated secondary antibodies for immunofluorescence were obtained from Jackson Immunoresearch (Cambridge House, St. Thomas Place, Ely CB7 4EX, UK), while goat anti-mouse and anti-rabbit Alexa Fluor^®^ 647-conjugated secondary antibodies for flow cytometry were from Cell Signaling Technology (Boston, MA, USA). DAPI was obtained from Biotium (Landing Parkway Fremont, CA, USA), while NucleoZOL was from Macherey-Nagel (Düren, Germany). dNTPs, primers, random hexamers, and PrimeScript reverse transcriptase were from Invitrogen (ThermoFischer Scientific, Waltham, MA, USA). Finally, the KAPA SYBR Fast Master Mix was obtained from Kapa Biosystems (Hoffmann-La Roche, Basel, Switzerland).

### 4.2. Cell Cultures

The HCE-2 cell line was obtained from American Type Culture Collection (ATCC, Middlesex, UK) and was cultured in a mixture (1:1) of Ham’s F-12 nutrient mixture and Dulbecco’s modified Eagle’s medium (DMEM/F12) supplemented with 15% FBS, 0.1 μg/mL cholera toxin, 0.5% DMSO, 5 μg/mL insulin, 40 μg/mL gentamycin, 10 ng/mL epidermal growth factor and 100 units/mL penicillin/streptomycin solution. HCE-2/ALDH3A1 and HCE-2/mock stable transfected cells [5,12] were cultured in the same medium in the presence of 0.2 mg/mL hygromycin B. The cells were maintained at 37 °C with 5% CO_2_ in a humidified incubator.

### 4.3. Analysis of Cellular Morphology

The cells were collected through trypsinization, resuspended in growth medium, washed with PBS, and finally analyzed with respect to their forward (FSC) and side scatter (SSC) profile in an Attune NxT flow cytometer. To quantify the morphological changes of HCE-2 cells concerning granularity, cellular size, and cell surface topography as a result of ALDH3A1 expression, the median fluorescence intensity values of FSC and SSC were determined with Flowjo software (BD Biosciences, Franklin Lakes, NJ, USA) [15]. The cells were also photographed by a ZEISS Primovert light microscope (Zeiss, Göttingen, Germany) equipped with a digital camera (Axiocam ERc 5 s by Zeiss, Oberkochen, Germany) under the 20× and 40× lenses.

### 4.4. Real-Time PCR

Real-time PCR was performed as previously described with minor modifications [5]. NucleoZOL reagent was used for isolating total RNA from cells according to the manufacturer’s instructions. Primers (Table 1) were designed with the Primer Express 3.0 software (Applied Biosystems, Waltham, MA, USA).

Total RNA (4.5 μg) was used for cDNA synthesis with the SuperScript™ First-Strand Synthesis System according to the manufacturer’s instructions. For Real-time PCR, KAPA SYBR Fast Master Mix was used according to the manufacturer’s instructions. The reactions were performed with an Applied Biosystems Step One instrument. The reactions were performed in triplicate, while data derived from three independent experiments were used for the final quantification of the gene expression ratio. *β-Actin* was used for normalizing gene expression according to the 2^−ΔΔCT^ method [46].

### 4.5. Scratch Assay

For the scratch assay, 7.5 × 10^5^ cells were seeded on 6-well plates a day prior to the experiment. Then, plates were washed twice with PBS and a straight scratch was introduced by drawing a sterilized pipette tip on the cellular confluent monolayer. The scratch closure was subsequently monitored every 12 h by a ZEISS Primovert light microscope (Zeiss, Göttingen, Germany) equipped with a digital camera (Axiocam ERc 5 s by Zeiss, Oberkochen, Germany). The acquired images were analyzed by ImageJ software (National Institute of Health, Bethesda, MD, USA) to estimate the average % percentage of gap closure determined as the change in gap width compared to the initial scratch.

### 4.6. Cell Counting and Growth Rate Determination

To investigate the growth rates of the isogenic cell line pair, 4 × 10^5^ HCE-2/mock or HCE-2/ALDH3A1 cells were plated on 10 cm culture dishes. The number of cells was determined at various time points for up to 84 h after staining with trypan blue using a Neubauer hemocytometer. The growth rate (the number of doublings occurring/day) and the doubling time were determined by the online calculator https://doubling-time.com/compute.php (accesed on 1 August 2022).

### 4.7. Cell Cycle Analysis

Approximately 1.5 × 10^6^ cells were seeded in 10 cm culture dishes and were placed in a humidified incubator (37 °C 5% CO_2_). Seeding was considered as t_0_ and then the cells were further cultured for different time points (16, 24, 36, 48, 72 h). At each time point, the cells were washed with PBS, fixed with ice-cold 75% ethanol, and incubated for at least 24 h at −20 °C. The cells were then washed with PBS, counted, suspended in RNase A (1 mg/mL), stained with propidium iodide (50 μg/mL), and incubated in the dark for 40 min. Finally, the % percentage of cells in each cell cycle phase was determined through flow cytometry analysis.

### 4.8. Apoptosis Analysis

Approximately 1.5 × 10^6^ of HCE-2/mock or HCE-2/ALDH3A1 cells were seeded on 10 cm culture dishes and placed in a humidified incubator (37 °C 5% CO_2_) a day prior to the experiment. Then, the cells were treated with either 200 μM H_2_O_2_ or 50 μM etoposide for 16 h. Subsequently, the early and late apoptotic percentage of cells was determined using an Annexin-FITC/PI apoptosis detection kit (BD Bioscience, San Jose, CA, USA) according to the manufacturer’s instructions. Briefly, the cells were collected with trypsin and washed twice with PBS. The cell pellet was then resuspended in Annexin V binding buffer to achieve a final concentration of 1 × 10^6^ cells per 1 mL of buffer. Subsequently, 100 μL of the cell suspension (1 × 10^5^ cells) was dyed with 5 μL of Annexin V and incubated for 13 min in the dark. Finally, the samples were stained with 5 μL of propidium iodide (PI) and analyzed by flow cytometry.

### 4.9. Immunofluorescence

**Figure 9:**
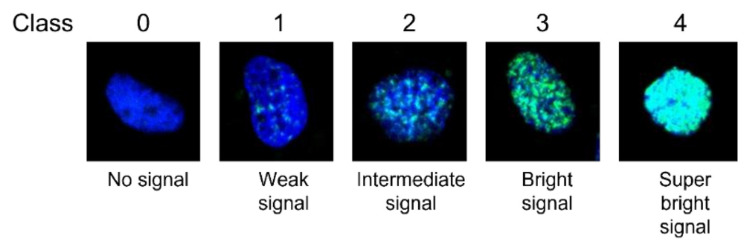
Representative classes of γH2AX foci levels (Alexa Fluor-488 intensity). Each cell was assigned a specific class (0: no signal 1: weak signal, 2: intermediate signal, 3: bright signal, or 4: superbright signal) according to Alexa Fluor 488 signal intensity corresponding to activated H2AX (γH2AX) levels.

Immunofluorescence was performed as previously described with few modifications [16]. Specifically, 1 × 10^5^ cells were grown in a monolayer on the surface of coverslips a day before the experiment. The cells were treated with either 200 μM H_2_O_2_ or 50 μM etoposide for 30 min and then washed with PBS and fixed with 4% formaldehyde in PBS for 20 min. Subsequently, the cells were washed with PBS (5 min, ×3) and paraformaldehyde was neutralized with the addition of 1 M of glycine (pH 8.5). The cells were permeabilized with 0.1% Triton Χ-100 in PBS for 5 min and blocking was performed by 5% (*w/v*) BSA in PBS for 5 min. Primary anti-γH2AX was used at a dilution of 1:500 (1 h, RT). The samples were then washed with PBS (×3) before incubation with Alexa Fluor^®^ 488-conjugated secondary antibody in PBS at a dilution of 1:400 (30 min, RT). Finally, the cells were stained with 4’,6-diamidino-2-phenylindole (DAPI) (1 μg/mL), washed, and finally mounted with MOWIOL (Calbiochem, Bad Soden, Germany). The samples were imaged with a 40×/NA 1.45 objective and an Andor Ixon + 885 digital camera on a customized Andor Revolution Spinning Disk Confocal System built around an IX81; Olympus stand (BioImaging-GR Facility, DUTH), while Andor IQ 2.7.1 software (Andor Technology, Belfast, UK) was used for image acquisition. Visualization of different samples was performed with the same exposure time and settings. To comparatively analyze the formation of γH2AX foci at the nucleus of cells, quantification methods were used, as previously described, with several modifications [47,48]. Specifically, each cell was scored as class 0 to 4 based on the intensity of Alexa Fluor-488 emission. Class 0 was representative of no signal, while class 4 was representative of a super bright signal (Figure 9).

For each sample, 100 cells were classified, while each cell was assigned a value based on its class. The overall score of each sample (total values of 100 cells) consequently ranged from 0 (100% in class 0) up to 400 (100% of cells in class 4). By this method, the intensity of γH2AX staining was expressed in arbitrary units.

### 4.10. Flow Cytometric Analysis of Total P53 and Phospho-p53 (Ser15)

To analyze the levels of total p53 and phospho-p53 (Ser15), cells were treated with either 200 μM H_2_O_2_ or 50 μM etoposide for 24 h. Then, the cells were collected through trypsinization, washed with PBS, and fixed with 4% formaldehyde at 25 °C for 15 min. Subsequently, they were washed once more with PBS and permeabilized with 100% ice-cold methanol for 10 min on ice. After permeabilization, the cells were washed (PBS) and immunostained with primary rabbit anti-p53 (1:800) or mouse anti-phospho-p53 (Ser15) (1:1600) antibodies for 1 h at room temperature. After an additional step of washing with PBS, the cells were stained with secondary goat anti-mouse or anti-rabbit AlexaFluor-647-conjugated (1:1000) antibody for 30 min at room temperature under dark conditions. Finally, the samples were washed and analyzed on the Attune NxT flow cytometer.

### 4.11. Western Immunoblotting

Western immunoblotting was performed as described previously with minor modifications [16]. To separate the nuclear and cytosolic parts, the cell pellets were lysed using a fractionation lysis buffer containing 10 mM HEPES, 10 mM KCl, 0.1 mM EDTA, 1.5 mM MgCl_2_, 0.2% NP-40 and supplemented with proteinase inhibitors (0.5 mM phenylmethylsulfonyl fluoride, 2.5 μg/mL aprotinin, 2.5 μg/mL pepstatin A, and 2.5 μg/mL leupeptin), and a phosphatase inhibitor cocktail. Cell lysates were incubated for 30 min at 4 °C, vortexed every 10 min, and centrifuged at 1000× *g* for 10 min to isolate the cytosolic (supernatant) and nuclear (pellet) fractions. Protein quantification was performed with a Pierce™ BCA Protein Assay kit, according to the manufacturer’s instructions.

Approximately 40 μg of cytosolic and 15 μg of nuclear samples were prepared and subjected to SDS-PAGE on Tris-Glycine gels. The resultant separated proteins were transferred to PVDF membranes while non-specific sites were blocked with 5% non-fat dry milk in TBST (150 mM NaCl, 100 mM Tris pH 7.5, and 0.1% *v/v* Tween-20), at room temperature for 2 h. The membranes were hybridized overnight at 4 °C with primary antibodies against ALDH3A1 (1:1000), H3 (1:1000), and β-tubulin (1:5000). Then, the membranes were incubated with secondary horseradish-peroxidase-conjugated anti-rabbit and anti-mouse IgG antibodies (1:5000) for 1 h at RT and immunoblot bands were developed through Chemidoc MD Imaging System (Biorad, Hercules, CA, USA). When necessary, blots were stripped with a stripping buffer (62.5 mM Tris-HCl pH 6.7, 2% SDS, 6 μL/mL 2-mercaptoethanol) and re-hybridized with an alternate antibody. Protein expression levels were quantified using NIH Image J software (National Institutes of Health, Bethesda, MD, USA), and the relative quantity of the protein with respect to *β*-tubulin or H3 protein was estimated.

### 4.12. Analysis of the Enzymatic Activity of ALDH3A1

The enzymatic activity of ALDH3A1 was determined in the cytosolic and nuclear extracts of the untreated HCE-2/ALDH3A1 cells as previously described [49]. A molar extinction coefficient of 6.22 mM^−1^/cm^−1^ was applied for NADPH, while the activity is expressed as the number of nmoles of NADPH produced per min and per mg of (total) protein when using benzaldehyde as a substrate.

### 4.13. Site-Directed Mutagenesis

Site-directed mutagenesis was utilized to introduce eight specific base pair substitutions altering the codons for lysine 265, lysine 266, lysine 277 and lysine 278 to alanines to “deactivate” the potential bipartite Nuclear Localization Signal (NLS) (265KKSLKEFYGEDAKKSRD281) [14] on the hALDH3A1 plasmid construct [16]. Site-directed mutagenesis was performed as previously described [12] via the Transformer TM kit by Clontech (TaKaRa Bio Inc., Kusatsu, Japa) according to the manufacturer’s instructions. Specifically, three oligonucleotides were synthesized. One acted as a selection primer, inducing the deactivation of the unique restriction site for NruI (underlined sequence) in the hALDH3A1 plasmid construct and two mutagenic primers were designed to introduce the desired base pair substitutions in the coding region of ALDH3A1 cDNA (underlined sequence): Selection primer: 5′-C AGC CTC GCG GCG CGC ACG CCA GCA AG-3′, Mutagenic primer A: 5′-CAA ATT GTG GAG AAG CTC GCG GCG TCA CTG AAA GAG TTC-3′ and Mutagenic primer B: 5′-C GGG GAA GAT GCT GCG GCA TCC CGG GAC TAT GGA AG-3′. Both the selection primer and the two mutagenic primers were simultaneously annealed to one strand of the denaturated hALDH3A1 plasmid DNA. Then, the mutated strand was further synthesized and ligated using T4 DNA polymerase and T4 DNA ligase. To ensure the selection of the constructs with the mutated NruI restriction site, digestion with NruI was performed. Subsequently, mutS *E. coli* bacteria (defective mismatch repair mechanism) were transformed with the digested plasmids. *E. coli* cells were then amplified and their plasmid DNA was isolated. The mixed plasmid pool was once more digested with NruI and re-transformed in mutS *E. coli* cells. Finally, single mutS *E. coli* colonies were amplified and their plasmid DNA was isolated and sequenced to confirm the Lys 265, Lys 266, Lys 277, and Lys 278 alterations (hALDH3A1_NLS_mut).

### 4.14. Transient Transfection

HCE-2 cells were transiently transfected with both hALDH3A1 (wild-type) and hALDH3A1_NLS_mut (mutated at the potent NLS region) constructs. For transient transfection, 2 × 10^5^ HCE-2 cells were plated on 10 cm culture plates a day prior to the experiment. Then, 16 μg of DNA and 40 μL of Lipofectamine reagent 3000 were mixed and added in 3 mL of Opti-MEM reduced serum medium and applied to the culture plates. After a 6 h incubation (37 °C, 5% CO_2_), Opti-MEM medium was replaced with fully supplemented DMEM-F12. The cells were then washed and the expression and cellular localization of ALDH3A1 were monitored by Western immunoblotting analysis.

### 4.15. Statistical Analysis

At least three independent experiments were performed for each sample per condition tested. All values are expressed as the mean ± S.D. GraphPad Prism software (version 8.3.0, GraphPad, Boston, MA, USA) was used for statistical analyses and graph construction. A comparison of the results between the two groups was performed by the Student’s *t*-test. An analysis of two variables among multiple groups was performed with a two-way ANOVA, followed by Tukey’s multiple comparison tests. A value of *p* < 0.05 was considered significant.

## 5. Conclusions

In conclusion, our data collectively support a cytoprotective role for ALDH3A1 in the corneal epithelium through multiple modes: (i) ALDH3A1 appears to promote the conversion of corneal epithelial cells towards a phenotype of low cell–cell adhesion, high migratory potential and low proliferation rate which may facilitate cell stress endurance, (ii) ALDH3A1 induces a lower proliferation rate in corneal epithelial cells, which is associated with an altered cell cycle progression and cell accumulation at the G2/M phase, (iii) ALDH3A1 elicits resistance to oxidative and genotoxic stress, which is accompanied by upregulation of p53 (both total and phospho-p53) and lower rates of H2AX foci formation, and (iv) the enhancement of p53 could be associated with a survival advantage under stress conditions by assisting in the more rapid and effective recruitment of the DDR machinery. Finally, the confirmation of the nuclear localization of the protein in HCE-2 cells implies novel, yet-uncharacterized ALDH3A1 functions, which warrant further investigation. Our study provides evidence supporting novel regulatory role(s) for ALDH3A1 through modulating various cellular pathways (differentiation, DDR, cell cycle, proliferation, and apoptosis) possibly by affecting the expression/regulation of p53, a key effector of cellular homeostasis. Further studies are required to elucidate the mechanistic details of the regulatory role(s) of ALDH3A1 in corneal epithelial homeostasis, which will greatly enable future translational studies and the development of more effective therapeutic interventions for corneal-related pathologies.

## Figures and Tables

**Table 1:** Primers used for the Real-time PCR comparative quantification studies.

Gene	Forward Primer	Reverse Primer
*β-actin*	GCGCGGCTACAGCTTCA	CTTAATGTCACGCACGATTTCC
*TJP-1*	CGACTCCTCGTCGGGTGAT	TTTGCTCCAACGAGATAATTTGG
*occludin*	CGAATCATTATGCACCAAGCAA	TGAGAGAGCATTGGTCGAACA
*claudin*	GGGAGGTGCCCTACTTTGCT	CCTTGGTGTTGGGTAAGAGGTT
*keratin12*	GATATGCGGGCGCAGTATG	ACCAGGCTTCAGCGTCCTT
*E-cadherin*	TACACTGCCCAGGAGCCAGA	TGGCACCAGTGTCCGGATTA
*N-cadherin*	CCTACTGGACGGTTCGCCAT	GTTGCAGTTGACTGAGGCGG
*vimentin*	TGAGTACCGGAGACAGGTGCAG	TAGCAGCTTCAACGGCAAAGTTC
*ZEB1*	CGAGTCAGATGCAGAAAATGAGCAA	ACCCAGACTGCGTCACATGTCTT
*ZEB2*	ACTATGGGGCCAGAAGCCAC	CTGCATGACCATCGCGTTCC
*CDK1*	AGGGTAGACACAAAACTACAGGTCAA	CCCTTCCTCTTCACTTTCTAGTCTGA
*CDK2*	GGAGCTTGTTATCGCAAATGC	TGCCTTGGCCGAAATCC
*CDK3*	CCCATGAGGTGGTGACACTGT	ATAGAACTTGCTGCCCAAGAGAA
*CDK4*	GATCTGATGCGCCAGTTTCTAA	CGGTGAACGATGCAATTGG
*p16INK4a*	CCCAACGCACCGAATAGTTAC	CGCTGCCCATCATCATGAC
*p27Kip1*	GGACTGCGGGACGATCCT	TGACAAGCCACGCAGTAGATTT
*p57Kip2*	GCGCGGCGATCAAGAA	TTGGCGAAGAAATCGGAGAT

## Data Availability

All data are contained within the article.

## References

[1] Koppaka V., Thompson D.C., Chen Y., Ellermann M., Nicolaou K.C., Juvonen R.O., Petersen D., Deitrich R.A., Hurley T.D., Vasiliou D.V. (2012). Aldehyde dehydrogenase inhibitors: A comprehensive review of the pharmacology, mechanism of action, substrate specificity, and clinical application. Pharmacol. Rev..

[2] Moreb J. (2008). Aldehyde Dehydrogenase as a Marker for Stem Cells. Curr. Stem Cell Res. Ther..

[3] Piatigorsky J. (2000). Review: A case for corneal crystallins. J. Ocul. Pharmacol. Ther..

[4] Evces S., Lindahl R. (1989). Characterization of rat cornea aldehyde dehydrogenase. Arch. Biochem. Biophys..

[5] Voulgaridou G.P., Tsochantaridis I., Tolkas C., Franco R., Giatromanolaki A., Panayiotidis M.I., Pappa A. (2020). Aldehyde dehydrogenase 3A1 confers oxidative stress resistance accompanied by altered DNA damage response in human corneal epithelial cells. Free Radic. Biol. Med..

[6] Lassen N., Bateman J.B., Estey T., Kuszak J.R., Nees D.W., Piatigorsky J., Duester G., Day B.J., Huang J., Hines L.M. (2007). Multiple and additive functions of ALDH3A1 and ALDH1A1: Cataract phenotype and ocular oxidative damage in Aldh3a1(-/-)/Aldh1a1(-/-) knock-out mice. J. Biol. Chem..

[7] Pappa A., Chen C., Koutalos Y., Townsend A.J., Vasiliou V. (2003). ALDH3A1 protects human corneal epithelial cells from ultraviolet- and 4-hydroxy-2-nonenal-induced oxidative damage. Free Radic. Biol. Med..

[8] Estey T., Piatigorsky J., Lassen N., Vasiliou V. (2007). ALDH3A1: A corneal crystallin with diverse functions. Exp. Eye Res..

[9] Pappa A., Estey T., Manzer R., Brown D., Vasiliou V. (2003). Human aldehyde dehydrogenase 3A1 (ALDH3A1): Biochemical characterization and immunohistochemical localization in the cornea. Biochem. J..

[10] Black W., Chen Y., Matsumoto A., Thompson D.C., Lassen N., Pappa A., Vasiliou V. (2012). Molecular mechanisms of ALDH3A1-mediated cellular protection against 4-hydroxy-2-nonenal. Free Radic. Biol. Med..

[11] Chen Y., Jester J.V., Anderson D.M., Marchitti S.A., Schey K.L., Thomson D.C., Vasiliou V. (2017). Corneal haze phenotype in *Aldh3a1*-null mice: In vivo confocal microscopy and tissue imaging mass spetrometry. Chem. Biol. Interact..

[12] Voulgaridou G.P., Tsochantaridis I., Mantso T., Franco R., Panayiotidis M.I., Pappa A. (2017). Human aldehyde dehydrogenase 3A1 (ALDH3A1) exhibits chaperone-like function. Int. J. Biochem. Cell Biol..

[13] Lassen N., Pappa A., Black W.J., Jester J.V., Day B.J., Min E., Vasiliou V. (2006). Antioxidant function of corneal ALDH3A1 in cultured stromal fibroblasts. Free Radic. Biol. Med..

[14] Pappa A., Brown D., Koutalos Y., DeGregori J., White C., Vasiliou V. (2005). Human aldehyde dehydrogenase 3A1 inhibits proliferation and promotes survival of human corneal epithelial cells. J. Biol. Chem..

[15] Jaroszeski M.J., Radcliff G. (1999). Fundamentals of flow cytometry. Mol. Biotechnol..

[16] Voulgaridou G.P., Kiziridou M., Mantso T., Chlichlia K., Galanis A., Koukourakis M.I., Franco R., Panayiotidis M.I., Pappa A. (2016). Aldehyde dehydrogenase 3A1 promotes multi-modality resistance and alters gene expression profile in human breast adenocarcinoma MCF-7 cells. Int. J. Biochem. Cell Biol..

[17] Kim H.N., Ruan Y., Ogana H., Kim Y.M. (2020). Cadherins, Selectins, and Integrins in CAM-DR in Leukemia. Front. Oncol..

[18] Siegel R.L., Miller K.D., Jemal A. (2020). Cancer statistics, 2020. CA. Cancer J. Clin..

[19] Mendonsa A.M., Na T.Y., Gumbiner B.M. (2018). E-cadherin in contact inhibition and cancer. Oncogene.

[20] Terzuoli E., Bellan C., Aversa S., Ciccone V., Morbidelli L., Giachetti A., Donnini S., Ziche M. (2019). ALDH3A1 overexpression in melanoma and lung tumors drives cancer stem cell expansion, impairing immune surveillance through enhanced PD-L1 output. Cancers.

[21] Wu D., Mou Y.P., Chen K., Cai J.Q., Zhou Y.C., Pan Y., Xu X.W., Zhou W., Gao J.Q., Chen D.W. (2016). Aldehyde dehydrogenase 3A1 is robustly upregulated in gastric cancer stem-like cells and associated with tumorigenesis. Int. J. Oncol..

[22] Zhang P., Sun Y., Ma L. (2015). ZEB1: At the crossroads of epithelial-mesenchymal transition, metastasis and therapy resistance. Cell Cycle.

[23] Vannier C., Mock K., Brabletz T., Driever W. (2013). Zeb1 Regulates E-cadherin and Epcam (Epithelial Cell Adhesion Molecule) Expression to Control Cell Behavior in Early Zebrafish Development. J. Biol. Chem..

[24] Mathow D., Chessa F., Rabionet M., Kaden S., Jennemann R., Sandhoff R., Gröne H.-J., Feuerborn A. (2015). Zeb1 affects epithelial cell adhesion by diverting glycosphingolipid metabolism. EMBO Rep..

[25] Feng Y., Feng Y., Zhu X., Dang Y., Ma Q. (2004). Alkali burn causes aldehyde dehydrogenase 3A1 (ALDH3A1) decrease in mouse cornea. Mol. Vis..

[26] Malumbres M., Barbacid M. (2009). Cell cycle, CDKs and cancer: A changing paradigm. Nat. Rev. Cancer.

[27] Besson A., Dowdy S.F., Roberts J.M. (2008). CDK inhibitors: Cell cycle regulators and beyond. Dev. Cell.

[28] Creff J., Besson A. (2020). Functional Versatility of the CDK Inhibitor p57Kip2. Front. Cell Dev. Biol..

[29] Qu Y., He Y., Yang Y., Li S., An W., Li Z., Wang X., Han Z., Qin L. (2020). ALDH3A1 acts as a prognostic biomarker and inhibits the epithelial mesenchymal transition of oral squamous cell carcinoma through IL-6/STAT3 signaling pathway. J. Cancer.

[30] Hande K.R. (1998). Etoposide: Four decades of development of a topoisomerase II inhibitor. Eur. J. Cancer.

[31] Collins A.R. (1999). Oxidative DNA damage, antioxidants, and cancer. BioEssays.

[32] Saiki J.P., Cao H., Van Wassenhove L.D., Viswanathan V., Bloomstein J., Nambiar D.K., Mattingly A.J., Jiang D., Chen C.H., Stevens M.C. (2018). Aldehyde dehydrogenase 3A1 activation prevents radiation-induced xerostomia by protecting salivary stem cells from toxic aldehydes. Proc. Natl. Acad. Sci. USA.

[33] Vasiliou V.K., Pappa A., Black W., Jester J., Day B., Min E., Lassen N. (2005). ALDH3A1 Prevents Apoptosis of Corneal Cells Induced by DNA Damaging Agents and Oxidative Stress. Invest. Ophthalmol. Vis. Sci..

[34] Meyer B., Voss K.O., Tobias F., Jakob B., Durante M., Taucher-Scholz G. (2013). Clustered DNA damage induces pan-nuclear H2AX phosphorylation mediated by ATM and DNA–PK. Nucleic Acids Res..

[35] Wu J., Clingen P.H., Spanswick V.J., Mellinas-Gomez M., Meyer T., Puzanov I., Jodrell D., Hochhauser D., Hartley J.A. (2013). γ-H2AX foci formation as a pharmacodynamic marker of DNA damage produced by DNA cross-linking agents: Results from 2 phase I clinical trials of SJG-136 (SG2000). Clin. Cancer Res..

[36] Jang J.H., Bruse S., Liu Y., Duffy V., Zhang C., Oyamada N., Randell S., Matsumoto A., Thompson D.C., Lin Y. (2014). Aldehyde Dehydrogenase 3a1 Protects Airway Epithelial Cells From Cigarette Smoke-Induced Dna Damage And Cytotoxicity. Free Radic. Biol. Med..

[37] Loughery J., Cox M., Smith L.M., Meek D.W. (2014). Critical role for p53-serine 15 phosphorylation in stimulating transactivation at p53-responsive promoters. Nucleic Acids Res..

[38] Chen J. (2016). The Cell-Cycle Arrest and Apoptotic Functions of p53 in Tumor Initiation and Progression. Cold Spring Harb. Perspect. Med..

[39] Lavin M.F., Gueven N. (2006). The complexity of p53 stabilization and activation. Cell Death Differ..

[40] Fan F., Yin R., Wang L., Zhao S., Lv D., Yang K., Geng S., Yang N., Zhang X., Wang H. (2021). ALDH3A1 driving tumor metastasis is mediated by p53/BAG1 in lung adenocarcinoma. J. Cancer.

[41] Koppaka V., Chen Y., Mehta G., Orlicky D.J., Thompson D.C., Jester J.V., Vasiliou V. (2016). ALDH3A1 Plays a Functional Role in Maintenance of Corneal Epithelial Homeostasis. PLoS ONE.

[42] Stagos D., Chen Y., Cantore M., Jester J.V., Vasiliou V. (2010). Corneal aldehyde dehydrogenases: Multiple functions and novel nuclear localization. Brain Res. Bull..

[43] Lu J., Wu T., Zhang B., Liu S., Song W., Qiao J., Ruan H. (2021). Types of nuclear localization signals and mechanisms of protein import into the nucleus. Cell Commun. Signal..

[44] Linsenmayer T.F., Beazley K.E., Cai C.X., Canner J.P., Fitch J.M., Kubilus J.K., Millholland J.M., Nurminskaya M., Talbot C., Zak N.B. (2015). Corneal Epithelial Nuclear Ferritin and Its Transporter Ferritoid Afford Unique Protection to DNA from UV Light and Reactive Oxygen Species. Studies on the Cornea and Lens. Oxidative Stress in Applied Basic Research and Clinical Practice.

[45] Alkhateeb A.A., Connor J.R. (2010). Nuclear ferritin: A new role for ferritin in cell biology. Biochim. Biophys. Acta Gen. Subj..

[46] Livak K.J., Schmittgen T.D. (2001). Analysis of Relative Gene Expression Data Using Real-Time Quantitative PCR and the 2^−ΔΔCT^ Method. Methods.

[47] Marti T.M., Hefner E., Feeney L., Natale V., Cleaver J.E. (2006). H2AX phosphorylation within the G1 phase after UV irradiation depends on nucleotide excision repair and not DNA double-strand breaks. Proc. Natl. Acad. Sci. USA.

[48] Khalaj M., Abbasi A., Yamanishi H., Akiyama K., Wakitani S., Kikuchi S., Hirose M., Yuzuriha M., Magari M., Degheidy H.A. (2014). A missense mutation in Rev7 disrupts formation of Polζ, impairing mouse development and repair of genotoxic agent-induced DNA lesions. J. Biol. Chem..

[49] Voulgaridou G.P., Mantso T., Chlichlia K., Panayiotidis M.I., Pappa A. (2013). Efficient E. coli Expression Strategies for Production of Soluble Human Crystallin ALDH3A1. PLoS ONE.

